# E2A Antagonizes PU.1 Activity through Inhibition of DNA Binding

**DOI:** 10.1155/2016/3983686

**Published:** 2016-01-28

**Authors:** Jason H. Rogers, Kristin S. Owens, Jeffrey Kurkewich, Nathan Klopfenstein, Sangeeta R. Iyer, M. Celeste Simon, Richard Dahl

**Affiliations:** ^1^Cancer Research and Treatment Center, University of New Mexico, Albuquerque, NM 87131, USA; ^2^Biological Science, University of Notre Dame, Notre Dame, IN 46556, USA; ^3^Department of Microbiology and Immunology, Indiana University School of Medicine, South Bend, IN 46617, USA; ^4^Abramson Family Cancer Research Institute, University of Pennsylvania, School of Medicine, Philadelphia, PA 19104, USA

## Abstract

Antagonistic interactions between transcription factors contribute to cell fate decisions made by multipotent hematopoietic progenitor cells. Concentration of the transcription factor PU.1 affects myeloid/lymphoid development with high levels of PU.1 directing myeloid cell fate acquisition at the expense of B cell differentiation. High levels of PU.1 may be required for myelopoiesis in order to overcome inhibition of its activity by transcription factors that promote B cell development. The B cell transcription factors, E2A and EBF, are necessary for commitment of multipotential progenitors and lymphoid primed multipotential progenitors to lymphocytes. In this report we hypothesized that factors required for early B cell commitment would bind to PU.1 and antagonize its ability to induce myeloid differentiation. We investigated whether E2A and/or EBF associate with PU.1. We observed that the E2A component, E47, but not EBF, directly binds to PU.1. Additionally E47 represses PU.1-dependent transactivation of the* MCSFR* promoter through antagonizing PU.1's ability to bind to DNA. Exogenous E47 expression in hematopoietic cells inhibits myeloid differentiation. Our data suggest that E2A antagonism of PU.1 activity contributes to its ability to commit multipotential hematopoietic progenitors to the lymphoid lineages.

## 1. Introduction

E2A, EBF, and Pax5 (BSAP) are early acting transcription factors regulating B lymphopoiesis [1]. Mice lacking any of these factors do not generate B cells with E2A and EBF deficiency blocking B lymphopoiesis at a slightly earlier stage of development than Pax5 deficiency [2–4]. Early B cell progenitors cell lines can be grown out from the fetal liver or bone marrow isolated from mice deficient in each of these factors. These lines all have the striking phenotype that besides being unable to differentiate into mature B cells they can be induced to differentiate into other hematopoietic lineages in vitro and in vivo [5–7]. Interestingly* E2A* ^−/−^ *, Ebf1* ^−/−^, and* Pax5* ^−/−^ pro-B cells all overexpress the myeloid cytokine receptor gene* MCSFR* (macrophage-colony stimulating factor receptor). Hematopoietic expression of* MCSFR* is dependent on the Ets family transcription factor PU.1 as demonstrated by the lack of detectable* MCSFR* mRNA in* PU.1* ^−/−^ hematopoietic cells [8, 9].

Mice lacking PU.1 do not make lymphocytes, myeloid cells, or the progenitors for these cells (CMPs, GMPs, or CLPs) [10, 11]. PU.1 activity is also critical for determining cell fate acquisition of uncommitted progenitors. High expression of PU.1 in hematopoietic progenitors directs myeloid development, and low expression directs B cell development [12]. Levels of PU.1 also have been shown to be important in the development of erythroid and myeloid cells [13]. PU.1 physically associates with the essential erythroid transcription factor, GATA1 [14]. The two factors mutually antagonize each other's transactivation potential. The ratio of GATA1 to PU.1 is important in determining whether an uncommitted precursor will develop into an erythroid or myeloid cell, respectively [15–19]. If PU.1 protein levels rise in an uncommitted cell, PU.1 continues to inhibit GATA1, and free PU.1 is able to activate genes required for myeloid differentiation.

Similar to GATA1, Pax5 binds to PU.1 and inhibits its ability to activate transcription [20]. However, Pax5 has a very limited ability to commit multipotential progenitors (MPPs) to early lymphoid progenitors, suggesting that, unlike GATA1, Pax5 does not repress PU.1 to promote B cell fate. Instead it likely functions to maintain the repression of myeloid genes in cells already committed to the B cell fate. EBF and E2A are necessary for the development of MPPs and lymphoid primed multipotential progenitors (LMPPs) into B lymphocytes [7, 21]. In our current study we examined whether E2A and/or EBF bind to PU.1 and antagonize its activity. The E2A protein E47, but not EBF, was observed to bind to PU.1 and block PU.1's association with DNA. This interaction inhibits PU.1 induced transcription from the* MCSFR* promoter and its induction of macrophage differentiation from hematopoietic progenitor cells. Our study suggests that E2A association with PU.1 may contribute to E2A's promotion of B lymphoid cell fate from multipotent hematopoietic progenitors.

## 2. Methods

### 2.1. In Vitro Binding Assays

Glutathione-S-transferase (GST) fusion proteins were prepared as previously described [22]. The GST-E47bHLH expression plasmid was generated by PCR. GST-PU.1, GST-E47bHLH, or GST bound to glutathione agarose were incubated with ^35^S-methionine labeled in vitro translated protein in 500 *μ*L of NETN buffer (20 mM Tris-HCl, pH 8.0, 200 mM NaCl, 1 mM EDTA, and 0.5% NP-40). In vitro translated products were prepared by the TNT reticulocyte lysate system (Promega). After 4 h incubation bound complexes were washed 4x in NETN buffer and eluted in sample buffer followed by separation by SDS-PAGE. Plasmids containing PU.1 deletion mutants and full-length Pax5 were obtained from Dr. M. Atchison (University of Pennsylvania) [20, 23]. Full-length human E47 was in vitro translated from the T7-E2/5 plasmid supplied by Dr. T. Kadesch (University of Pennsylvania). Truncations CT1 and CT2 were generated by linearizing T7-E2/5 plasmid with NotI and XhoI, respectively, before in vitro transcription/translation reaction. The amino terminal E47 truncations were generated by PCR. Human EBF protein was in vitro translated from pSport-EBF obtained from ATCC. ARNT protein was in vitro translated from pcDNA3-ARNT, which was provided by Dr. B. Keith (University of Pennsylvania).

### 2.2. Coimmunoprecipitations

Nuclear extracts were prepared from K562 cells. 2 mg of nuclear extract was incubated in binding buffer (20 mM Hepes, 25% glycerol, 10 mM KCl, 1.5 mM MgCl_2_, 0.2 mM EDTA, and 0.5 mM DTT) with 1 *μ*g anti-PU.1 or 1 *μ*g anti-GMCSFR*α* (Santa Cruz Biotechnology, sc-352 and sc-691) antibody prebound to protein A agarose beads overnight at 4°C. Agarose beads and captured protein complexes were washed 5X in binding buffer. Protein lysates were eluted in SDS-PAGE sample buffer.

### 2.3. Immunoblotting

Immunoblots were performed by resolving protein lysates via SDS-PAGE and transferring to nitrocellulose membrane (Gibco-BRL). The membranes were incubated with indicated antibodies and anti-mouse or anti-rabbit (Cell Signaling) horseradish peroxidase- (HRP-) conjugated secondary antibodies. Immunoreactive bands were detected using Supersignal (Pierce). Anti-E47 (Cat. #554077) and anti-PU.1 (Cat. #554268) were obtained from BD Biosciences.

### 2.4. Reporter Constructs and Expression Plasmids

The −74 to +67 141 bp human* GCSFR* promoter and −416 to +124 human* MCSFR* promoter were PCR amplified with previously described primer sets [24, 25] and cloned into pGL3 Basic (Promega). The pcDNA3-PU.1, MigR1-C/EBP alpha, MigR1-PU.1, (GAL4) 5-luciferase plasmid, and GAL4-PU.1 fusion plasmids have been previously described [22, 26]. The MigR1-E47 and (*μ*E3)_4_-Luciferase plasmids were provided by Drs. W. Pear and T. Kadesch (University of Pennsylvania). (*μ*E3)_4_-Luciferase contains 4 E box sites from the *μ* heavy chain locus upstream of the luciferase gene.

### 2.5. Transfections

U937 cells were electroporated as previously described [27] using 25 *μ*g of luciferase reporter plasmid, 50 *μ*g MigR1-E47 plasmid, and 5 *μ*g of the thymidine kinase promoter renilla luciferase (pRL-tk) plasmid. 293 T cells were transfected using the BD CalPhos mammalian transfection kit (BD Biosciences). For* MCSFR* reporter assays, cells were cotransfected with 2 *μ*g pGL3-MR, 4 *μ*g MigR1-PU.1, 4 *μ*g MigR1-E47, and 25 ng of pRL-tk. For E2A-reporter assays, cells were transfected with 2 *μ*g (*μ*E3)_4_-Luciferase, 4 *μ*g MigR1-PU.1 and/or 4 *μ*g MigR1-E47, and 25 ng of pRL-tk. Total amount of plasmid was kept constant with MigR1 plasmid. 48 h after transfection cell lysates were harvested using Promega cell lysis buffer. Firefly and renilla luciferase activity were measured using the Dual-Luciferase Assay System (Promega). Luciferase values were normalized to renilla luciferase values in order to adjust for potential differences in transfection efficiency.

### 2.6. Retroviral Transduction of PUER Cells

Retroviral vectors, MigR1 or MigR1-E47, were cotransfected into 293 T cells together with the retroviral packaging vector pCL-Eco (Imgenex) using calcium phosphate precipitation. 48 h after transfection retroviral supernatants were harvested. PUER progenitor cells were infected by resuspending in retroviral supernatant containing polybrene (8 *μ*g/mL) and centrifuging at 2000 ×g for 2 h at 25°C. After the spin infection, the cells were recultured in fresh media containing rIL-3. Cell lines expressing high levels of GFP were obtained by limiting dilution cloning.

### 2.7. PUER Differentiation

The generation of PUER progenitor cells and their differentiation into macrophages is described [26, 28]. Cells were maintained in IMDM, 10% fetal calf serum (Hyclone), 1 U/mL penicillin/streptomycin, 2 mM L-glutamine, and 50 mM beta-mercaptoethanol. Media contained 5 ng/mL IL-3 (R&D systems). Cells were differentiated by adding 100 nM of OHT to the media. Immunohistochemistry was performed as previously described [29].

### 2.8. Primary Hematopoietic Cell Differentiation

The use of mice in these experiments was approved by the University of New Mexico LACUC (Protocol #07UNM027). In vitro culture of lineage negative bone marrow cells was performed as previously described [30]. Lineage negative bone marrow cells prepared with MACS lineage cell separation kit (Miltenyi Biotec). Cells were infected with MigR1 or MigR1-E47 retroviral supernatant. After infection cells were cocultured with OP9 cells in IMDM media containing 1 ng/mL IL-7 and 5 ng/mL Flt3L. After 12 d of coculture differentiation cells were stained with CD19-PE and CD11b-APC (Invitrogen) and analyzed with a FacsCalibur flow cytometer (Becton Dickinson).

### 2.9. Electrophoretic Mobility Shift Assay and Chromatin Immunoprecipitation

Nuclear extracts were prepared from GFP or E47 expressing PUER cell lines as described [31]. Nuclear extract were also prepared from 293 T cells transfected by calcium phosphate with PU.1 and/or E47 expression plasmids. 5 *μ*g of nuclear extract was incubated with 10,000 counts per minute (cpm) of end-labeled oligonucleotide probe in binding buffer (7.5 mM Tris-HCl (pH 7.5), 0.74 mM EDTA, 3% ficoll, 56 mM KCl, and 0.75 mM DTT). It was indicated that 100 *μ*g of unlabled wildtype or mutant competitor oligonucleotide was added to binding reactions to demonstrate specific DNA binding. Additionally it was indicated that 2 *μ*l of rabbit anti-PU.1 antibody was added to binding reactions to determine if PU.1 was contained in DNA/protein complexes. After a 30-minute incubation at room temperature samples were separated on an 8% nondenaturing polyacrylamide gel. Oligonucleotides used for EMSA analysis were derived from the human MCSFR promoter, WT MCSFR: cctagctaaaaggggaagaagaggatcagc, MT MCSFR: cctagctaaaagggatcggtaccgatcagc. Underlined nucleotides indicate differences between wildtype and mutant oligonucleotides.

Chromatin immunoprecipitations were performed as described [32]. Chromatin preparation was incubated with normal rabbit serum, anti-PU.1 antibody (sc-352, Santa Cruz Biotechnology), prebound to protein A/G agarose. An aliquot of chromatin was saved for input. Immunoprecipitates were washed, crosslinks were reversed, and protein was digested with proteinase K. Isolated DNA was purified and DNA pellets were resuspended in sterile H_2_O. The MCSFR promoter was amplified by PCR.

## 3. Results

### 3.1. PU.1 Interacts with the E2A Subunit E47 but Not with EBF

**Figure 1:**
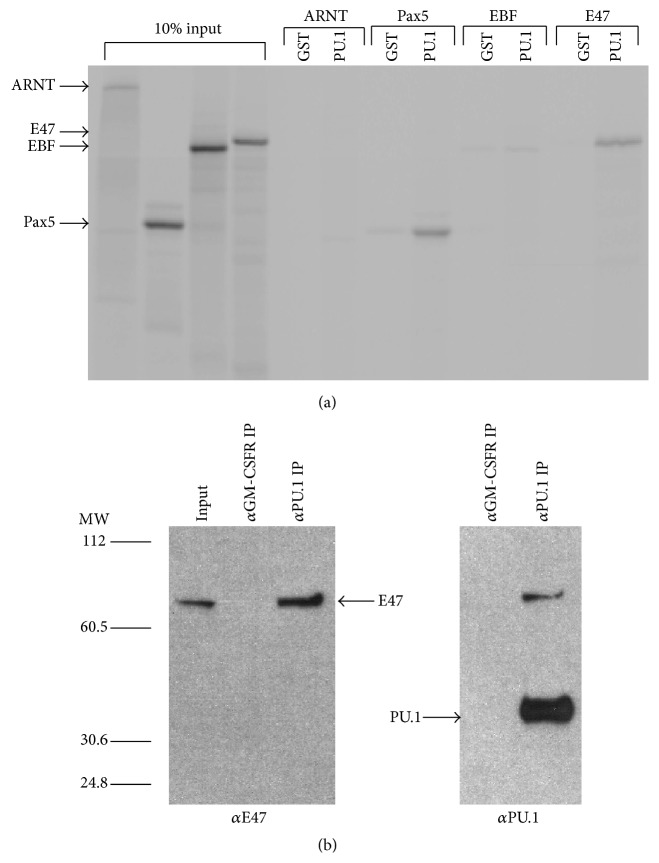
E47 but not EBF interacts with PU.1. (a) The transcription factors ARNT, PAX-5, EBF, and E47 were in vitro translated (IVT) and labeled with ^35^S-methionine. The labeled proteins were incubated with either GST or GST-PU.1 bacterially produced protein. 10% of IVT proteins and proteins bound to GST and GST-PU.1 were separated by SDS-PAGE and visualized by autoradiography. ARNT is a basic-helix-loop-helix transcription factor, which was used as a negative control (not expected to bind to PU.1). Pax-5 was used as a positive control. (b) E47 and PU.1 are coexpressed in the human hematopoietic cell line K562. Nuclear extracts were prepared from K562 cells and treated with anti-PU.1 antibody or anti-GM-CSFR*α* prebound to protein A agarose. Isolated complexes were separated on an SDS-PAGE gel and blotted. The presence of E47 in complexes was assayed using an anti-E47 antibody.

In vitro binding assays were performed to determine if E2A and/or EBF bind to PU.1. In vitro translated Pax5 (positive control), ARNT (negative control), EBF and the E2A subunit E47 were incubated with either purified GST or GST-PU.1 fusion protein. In agreement with previous data, Pax5 bound to GST-PU.1, but not GST (Figure 1(a)) [20]. EBF did not bind significantly to either protein; however, E47 specifically interacted with GST-PU.1 (Figure 1(a)). To demonstrate that this was not a nonspecific interaction between the basic domain of E47 and PU.1, the basic helix-loop-helix PAS domain protein ARNT was incubated with GST-PU.1 and no binding was detected [33].

To determine if E47 and PU.1 interacted in vivo, nuclear extracts were prepared from the K562 hematopoietic cell line, which coexpresses both PU.1 and E47 endogenously. Extracts were incubated with anti-PU.1 or anti-GMSCFR*α* antibody prebound to protein A agarose beads. E47 was detected in PU.1 immunoprecipitates, but not in anti-GMCSFR immunoprecipitates (Figure 1(b)). These data demonstrate that PU.1 and E47 associate both in vitro and in vivo.

**Figure 2:**
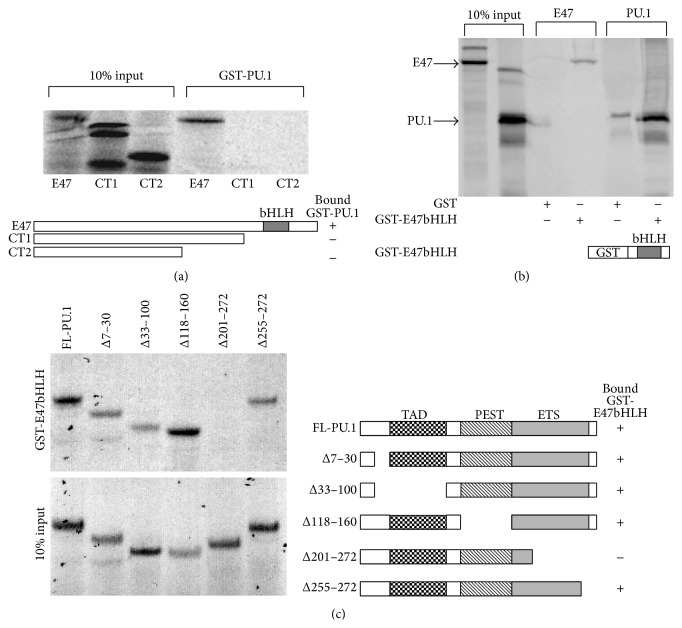
Identification of domains required for mediating the interaction between PU.1 and E47. (a) In vitro translated ^35^S-methionine labeled full-length human E47 (FL, aa 1–654), E47 C-terminal truncation 1 (CT1, aa 1–492), and E47 C-terminal truncation 2 (CT2, aa 1–369) were incubated with bacterially produced GST-PU.1 fusion protein. 10% input proteins and proteins bound to GST-PU.1 were separated by SDS-PAGE and visualized by autoradiography. (b) ^35^S-methionine labeled in vitro translated PU.1 and E47 were incubated with GST-E47bHLH protein (E47, aa 521–623). In vitro translated E47 was used as a positive control since it should dimerize with the E47 bHLH domain. (c) ^35^S-methionine labeled in vitro translated PU.1 deletion mutants were incubated with GST-E47bHLH protein.

To delineate regions of both proteins that mediate the interaction, further in vitro binding assays were performed. In vitro translated full-length E47 and E47 truncation mutants were incubated with GST-PU.1. As shown previously, full-length E47 bound to the GST-PU.1 protein. However, neither of the two carboxyl terminal deletion mutants (CT1, CT2) lacking the basic-helix-loop-helix (bHLH) DNA binding domain could bind to PU.1 (Figure 2(a)). This suggested that the bHLH domain of E47 mediated the interaction with PU.1. To confirm this conclusion, a GST-E47bHLH fusion protein was prepared and incubated with either in vitro translated E47 or PU.1. As expected the E47 bHLH domain mediated homodimerization with E47. The bHLH domain also pulled down PU.1 demonstrating that it is sufficient for mediating the interaction with PU.1 (Figure 2(b)).

To identify the domain of PU.1 that interacts with E47, in vitro translated deletion products of PU.1 were incubated with purified GST-E47bHLH protein. Deletion of the transactivation domain (Δ33–100) and the PEST region (Δ118–160) of PU.1 had no effect on binding to E47. However, a deletion construct lacking the Ets DNA binding domain (Δ201–272) was unable to bind to E47 (Figure 2(c)). These data demonstrate that PU.1 and E47 bind to each other through their DNA binding domains.

### 3.2. E47 Inhibits PU.1 Induced Transcription

**Figure 3:**
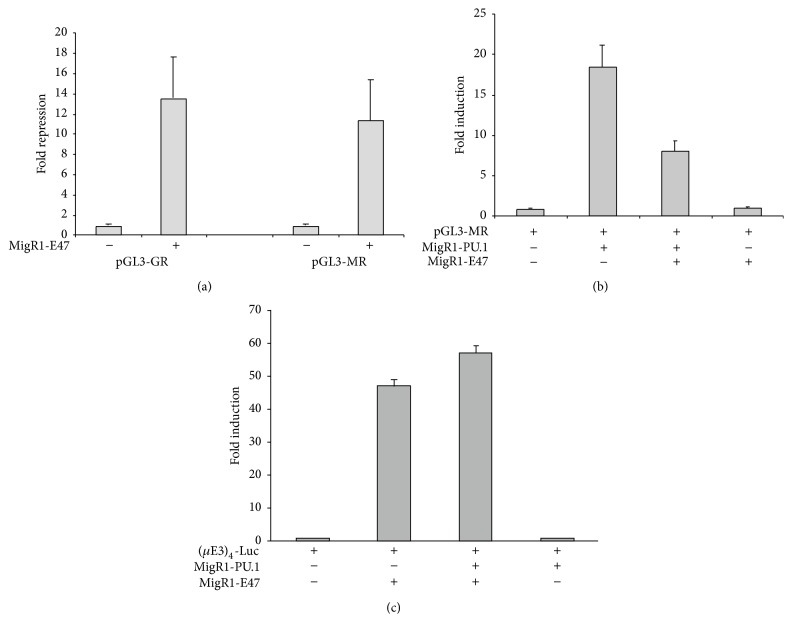
E47 inhibits promoter activity induced by PU.1. (a) Transient transfection of U937 cells with a* MCSFR* promoter and* GCSFR* promoter luciferase construct in the presence or absence of MigR1-E47 plasmid. (b)* MCSFR* promoter activity induced in 293 T cells with cotransfection of MigR1-PU.1 in the presence or absence of MigR1-E47 plasmid. MigR1-E47 by itself had no effect on* MCSFR* promoter activity. (c) (*μ*E3)_4_-Luciferase activity induced by cotransfection with MigR1-E47 in the presence and absence of MigR1-PU.1. For (a) luciferase activity is reported as fold repression compared to activity in the absence of MigR1-E47. For (b) and (c) luciferase activity is reported as fold-induction above the activity seen in 293 T cells transfected with only the reporter plasmid and MigR1. Luciferase values are the mean ± standard deviation of three independent transfections.

To investigate whether the interaction between PU.1 and E47 had any functional consequences, transient transfection reporter assays in U937 myeloid cells were performed. Two PU.1-dependent promoters were initially examined: the* GCSFR* and* MCSFR* promoters [24, 34]. E47 expression decreased expression from both the* MCSFR* and* GCSFR* promoter constructs over 10-fold (Figure 3(a)). Further experiments were carried out with the* MCSFR* promoter construct to determine if this repression was specifically targeting PU.1 activity. When the reporter was cotransfected into 293 T cells with PU.1 there was over 15-fold activation of reporter expression above that seen in the absence of PU.1 (Figure 3(b)). However, when E47 was expressed along with PU.1,* MCSFR* promoter activity was less than half that observed with PU.1 alone. E47 had no significant effect on the transcription of the* MCSFR* reporter plasmid in the absence of PU.1. We also determined whether PU.1 could repress E47 (E2A) induced transcriptional activity. Cells cotransfected with an E2A-reporter plasmid ((*μ*E3)_4_-Luciferase) and E47 expression plasmid resulted in an almost 50-fold enhancement of luciferase activity over cells transfected with the E2A-reporter alone. Coexpression of PU.1 with E47 did not repress transcriptional activity from the reporter construct (Figure 3(c)).

### 3.3. PU.1 Induced Myeloid Differentiation Is Blocked by Exogenous E47 Expression

**Figure 4:**
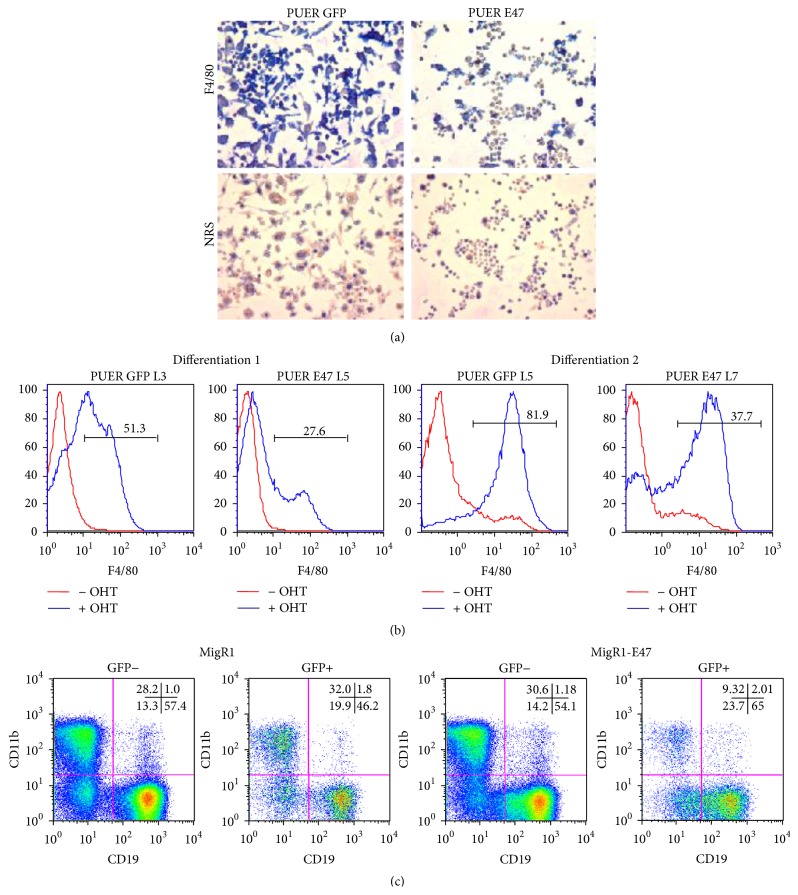
E47 inhibits myeloid differentiation of hematopoietic cells. (a) PUER cells were superinfected with MigR1 or MigR1-E47 virus and pure GFP expressing clones generated. Cell lines were either undifferentiated (no OHT) or differentiated (100 nM OHT) for 8 d. Immunohistochemistry of adherent PUER cell lines induced to differentiate with 100 nM OHT for 8 d. Cells were stained either with the macrophage differentiation marker F4/80 or with normal rat serum (NRS). Magnification is 400x. (b) F4/80 surface expression as determined by flow cytometry of independent PUER GFP and PUER E47 lines with no addition of OHT and after 8 d of OHT treatment. (c) Flow cytometry analysis of lineage minus hematopoietic progenitors infected with control MigR1 virus or MigR1-E47 virus. Cells were cultured for 12 d on OP9 stromal cell layer in the presence of 1 ng/mL IL-7 and 5 ng/mL Flt3L. Differentiation was analyzed by staining cells with anti-CD11b (myeloid) and anti-CD19 (B cell). Percentages of total cells positive for CD11b and CD19 expression are shown in the upper right corner of FACs plots.

We tested the ability of E47 to affect PU.1's biological activity by assaying the differentiation of the PUER myeloid progenitor cell line [28]. These cells are derived from the fetal liver of* PU.1* ^−/−^ mice and express a fusion protein between PU.1 and the estrogen receptor ligand-binding domain. In the absence of the ligand hydroxytamoxifen (OHT), these cells remain undifferentiated. In the presence of 100 nM OHT, PUER cells differentiate into macrophages. These cells were superinfected with either an E47-IRES-GFP virus or a GFP only virus. Cell lines expressing high levels of GFP were generated and their ability to differentiate into macrophages was assayed. After 8 d of OHT treatment, PUER GFP cultures became adherent and expressed the macrophage specific marker F4/80 (Figure 4(a)). In contrast the majority of PUER E47 cells remained nonadherent and were predominantly negative for F4/80. Additionally we analyzed surface expression of F4/80 by flow cytometry. Two separate differentiations were examined with 2 independent PUER GFP and PUER E47 cell lines before and after 8 d of culture with OHT to induce PU.1 activity. In both differentiations we observed that E47 decreased the expression of F4/80 (Figure 4(b)) similar to what was observed with the immunohistochemistry results.

To determine if E47 inhibited myeloid differentiation of primary cells, lineage negative hematopoietic progenitors were isolated and infected with control GFP or E47-IRES-GFP retroviruses. To assay myeloid versus lymphoid differentiation, the infected progenitors were cultured on an OP9 stromal cell layer in the presence of IL-7 and Flt3L cytokines. These culture conditions strongly promote B cell development but are also permissive for myeloid development. After 12 d of culture, differentiation was assayed by cell surface expression of the myeloid specific protein CD11b and B cell specific protein CD19 (Figure 4(c)). In the control (GFP only virus) cultures both the uninfected and the infected (GFP+) populations contained approximately 30% CD11b+ cells. In the E47 retrovirus treated cultures the uninfected GFP− population also contained approximately 30% CD11b+ cells, but in the infected GFP+ population only 10% of the cells were CD11b+, which is similar to the inhibition of myeloid development seen with the PUER cell line (Figures 4(a) and 4(b)). The E47 infected population also had an increased percentage of CD19+ B cells.

### 3.4. E47 Abrogates PU.1 DNA Binding

**Figure 5:**
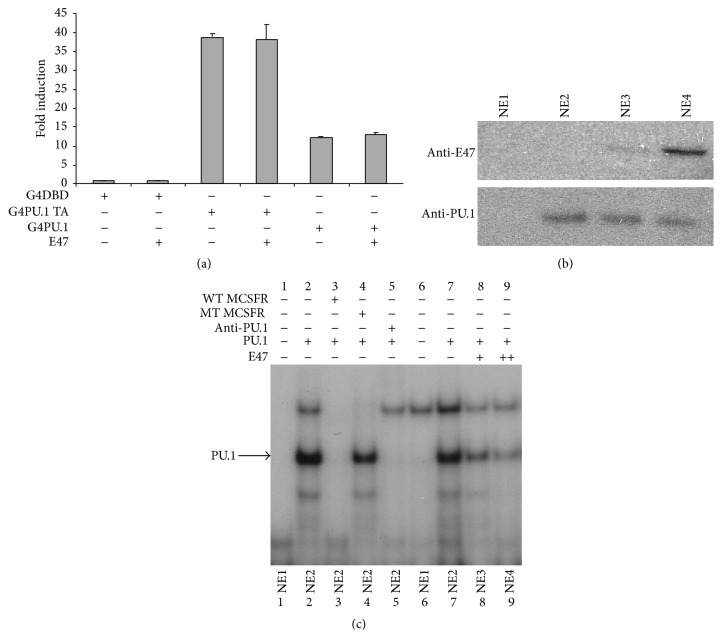
E47 inhibits PU.1 binding to DNA. (a) Transient transfections of 293 T cells with a GAL4 responsive luciferase reporter construct. Cells were cotransfected with either GAL4 full-length PU.1 expression plasmid or GAL4-PU.1 activation domain in the presence or absence of MigR1-E47 plasmid. Luciferase activity is the mean ± standard deviation of three independent transfections and luciferase activity is reported as fold-induction above the activity seen in 293 T cells transfected with the GAL4 DNA binding domain expression plasmid. (b) Immunoblot of nuclear extracts prepared from 293 T cells transfected with empty expression plasmids (NE1), PU.1 expression plasmid (NE2), and PU.1 and E47 expression plasmids (NE3, NE4). NE4 was prepared from cells transfected with an increased amount of E47 expression plasmid (++). Immunoblots were probed with anti-PU.1 or anti-E47 antibody as indicated. (c) EMSA performed with nuclear extracts from (b) and a ^32^P-labeled* MCSFR* probe. The PU.1 containing complex was identified by performing competitions with unlabeled wildtype and mutant* MCSFR* probes as indicated. Additionally the PU.1 containing complex could be ablated by inclusion of anti-PU.1 antibody in the binding mix.

Since E47 binds to the DNA binding domain of PU.1, we determined if E47 represses PU.1 activity if DNA binding was not dependent on the Ets domain. Full-length PU.1 and the PU.1 transactivation domain alone were fused to the GAL4 DNA binding domain and transactivation activity of the hybrid proteins was assayed by transient transfection of 293 T cells with a GAL4 reporter plasmid. Both fusion proteins activated luciferase expression. The full-length PU.1 fused to GAL4 did not activate transcription as robustly as the PU.1 transactivation domain alone, GAL4 fusion, potentially due to the Ets domain in the full-length allowing the fusion to be competed from the GAL4 reporter plasmid by Ets sites in the genome. Importantly though, neither fusion construct was inhibited by E47 (Figure 5(a)). This result suggested that E47 represses PU.1 activity by interfering with PU.1's ability to bind to DNA.

To test this hypothesis we assayed PU.1 DNA binding in nuclear extracts prepared from 293 T cells transfected with PU.1 and/or E47 expression plasmids. Nuclear extracts were incubated with a labeled* MCSFR* promoter fragment (Figures 5(b) and 5(c)). We identified protein complex that was competed away by unlabeled wildtype* MCSFR *probe (lane 3) but not a similar probe containing a mutated PU.1 binding site (lane 4) from the extracts expressing only PU.1. This complex was ablated by addition of a PU.1 antibody (lane 5). In nuclear extracts coexpressing PU.1 and increasing amounts of E47 we observed a decrease in the PU.1/*MCSFR* complex (lanes 8, 9). Expression of PU.1 and E47 in the 293 T extracts is shown by immunoblot (Figure 5(b)).

**Figure 6:**
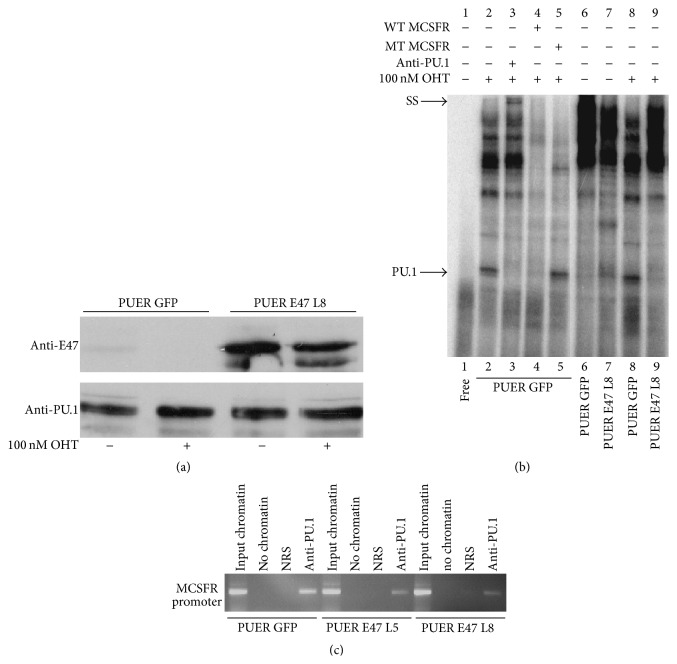
E47 blocks PU.1 from binding to DNA in hematopoietic cells. (a) Immunoblot of PUER and E47 protein expression in nuclear extracts prepared from PUER GFP and PUER E47 cell lines which were grown in the absence or presence (+) of 100 nM OHT. (b) Electrophoretic mobility shift assay (EMSA) with a ^32^P-labeled DNA probe from the* MCSFR* promoter. Nuclear extracts were prepared from PUER GFP and PUER E47 cell lines that were grown for 2 d in the absence or presence (+) of 100 nM OHT. SS indicates supershifted complex. (c) Chromatin immunoprecipitation (ChIP) of the* MCSFR* promoter from PUER GFP and 2 independent PUER E47 cell lines using anti-PU.1 antibody. PUER cell lines were treated for 7 d with OHT. Chromatin also precipitated with normal rabbit serum (NRS) to demonstrate specificity of the anti-PU.1 immunoprecipitations.

To determine if E47 abrogates PU.1 DNA binding in hematopoietic cells we performed EMSAs with GFP− and E47− expressing PUER cell nuclear extracts. As shown by immunoblot E47-infected cells expressed substantially more E47 in the presence or absence of OHT, than GFP-infected PUER (Figure 6(a)). As done previously a PU.1 containing DNA binding complex was identified in the OHT-treated PUER GFP cells using unlabeled wildtype and mutant* MCSFR* probes. This complex was supershifted (indicated by SS) by a PU.1 antibody (lane 3). Additionally this complex was induced by OHT treatment as it was not present in extracts from untreated PUER GFP cells (lane 6). Significantly the PU.1 containing complex was not detectable in PUER E47 OHT-treated cells (Lane 9).

Using PUER GFP cells and two independent lines of PUER E47 cells, chromatin immunoprecipitations (ChIP) with anti-PU.1 were performed to determine if E47 would antagonize PU.1 DNA binding in cells. Anti-PU.1 efficiently precipitated the MCSFR promoter, whereas the promoter was not detected in normal rabbit serum immunoprecipitates. Consistent with our findings with the EMSA assays, less MCSFR promoter was precipitated with anti-PU.1 from chromatin isolated from both PUER lines expressing E47 (Figure 6(c)). The results of EMSAs and ChIP assays support the conclusion that E47 inhibits the ability of PU.1 to bind to DNA.

## 4. Discussion

We have demonstrated that the E2A protein E47 binds to PU.1 and blocks its ability to bind to DNA. This inhibition of DNA binding resulted in repression of* MCSFR* promoter activity and inhibited myeloid differentiation. The E47 bHLH domain mediates the interaction with PU.1, so potentially other bHLH proteins may also be able to bind to PU.1 and affect its activity. Preliminary results demonstrated that another E2A related molecule HEB binds to PU.1 (data not shown). However, there is specificity to the binding of PU.1 to bHLH proteins as the bHLH PAS domain protein, ARNT, cannot bind to PU.1 (Figure 1(a)). The Ets family member, Ets1, has been shown to bind to the bHLH factor USF1 through interactions between their respective DNA binding domains similar to what we describe for PU.1 and E2A [35]. However, in the case of Ets1 and USF1 the interaction enhances the pairs DNA binding and transactivation of target genes. Interestingly PU.1 was shown not to interact with USF1, demonstrating that there is specificity in the binding of bHLH and Ets family members. Since PU.1 binds to the DNA binding domain of E47 it is somewhat surprising that PU.1 is unable to block E47 binding to DNA. However, it has been shown that PU.1 binds to the DNA binding domain of GATA1, without affecting GATA1's association with DNA [19], and similarly c-Jun, GATA1, C/EBP alpha, and Pax5 bind to the Ets domain of PU.1 without disrupting PU.1's association with DNA [17, 18, 20, 36].

E47 inhibited macrophage differentiation of PUER cells and myeloid differentiation of primary hematopoietic cells suggesting that E47 may play a role in cell fate decisions. This may explain why high levels of PU.1 are required for inducing myeloid differentiation [12]. In an uncommitted progenitor cell there may need to be sufficient PU.1 protein to bind to E47 and other inhibitors yet still leave enough free PU.1 protein available for binding to the regulatory regions of myeloid target genes. By stochastic mechanisms or through cytokine signaling one factor may increase in concentration in relation to the other and commit cells to either myeloid (PU.1) or B cell (E2A) differentiation. Unlike in the cases of GATA1 we did not observe a coordinate ability of PU.1 to repress E2A driven transcription (Figure 3(c)). However, overexpression of PU.1 in early CD4−CD8− double negative thymocytes and induction of PU.1 in PUER cells have demonstrated that PU.1 upregulates the expression of the bHLH inhibitor Id-2 [37, 38]. A similar upregulation of Id-2 by PU.1 in early hematopoietic progenitors may be an indirect mechanism by which PU.1 represses E2A activity. There is evidence that E2A specifies hematopoietic cell fate of multipotent cells. 70Z/3 pre-B lymphocytes can be converted to macrophages and this is associated with the loss of E2A and EBF expression. If the E2A protein E12 is expressed in 70Z/3 macrophages, they are converted to B cells [39]. An analysis of LMPPs isolated from* E2A* ^−/−^ mice showed that the lack of E2A results in increased development of monocytes and granulocytes from these progenitors [21]. Similarly ectopic expression of the E2A inhibitor, Id1, in LMPPs promotes myeloid differentiation [40].

Consistent with our observations Bhalla et al. previously observed that E2A component E12 could induce B cell differentiation of fetal liver MPPs [41]. E2A activity can be heterodimers or homodimers of E12 and/or E47. The two proteins are products of alternatively spliced transcripts generated from the same gene (Tcf3) that differ by the use of two exons that encode alternative bHLH domains. The distinct bHLH domains of E12 and E47, however, share >80% amino acid identity and interact with the same sets of proteins [42]. Interestingly the Bhalla report showed that E2A inhibition of macrophage development required the transcription factor's transactivation domain. Since we observe that the E2A DNA binding domain mediates the interaction between PU.1 and E2A this might suggest that other non-PU.1 functions may be needed to inhibit macrophage development. Alternatively the activation domain may not be required for direct binding to PU.1 but may still be needed to inhibit PU.1's DNA binding and/or stabilize the PU.1/ E2A interaction.

## 5. Conclusions

Our data demonstrates that E2A and PU.1 directly bind to each other, which results in antagonism of PU.1's DNA binding activity. Previous inhibitory binding partners of PU.1 such as Pax5, GATA1, and C/EBP alpha have either recruited in transcriptional corepressors or displaced coactivators without affecting association with DNA. The observations support the hypothesis that during hematopoiesis E47 can antagonize PU.1 activity to direct B cell development. If PU.1 levels are high though, PU.1 overcomes this antagonism and directs myeloid differentiation.
